# Proton Magnetic Resonance Spectroscopy Reveals Neuroprotection by Oral Minocycline in a Nonhuman Primate Model of Accelerated NeuroAIDS

**DOI:** 10.1371/journal.pone.0010523

**Published:** 2010-05-07

**Authors:** Eva-Maria Ratai, Jeffrey P. Bombardier, Chan-Gyu Joo, Lakshmanan Annamalai, Tricia H. Burdo, Jennifer Campbell, Robert Fell, Reza Hakimelahi, Julian He, Patrick Autissier, Margaret R. Lentz, Elkan F. Halpern, Eliezer Masliah, Kenneth C. Williams, Susan V. Westmoreland, R. Gilberto González

**Affiliations:** 1 A. A. Martinos Center for Biomedical Imaging and Neuroradiology Division, Massachusetts General Hospital, Charlestown, Massachusetts, United States of America; 2 Harvard Medical School, Boston, Massachusetts, United States of America; 3 Division of Comparative Pathology, New England Regional Primate Research Center, Southborough, Massachusetts, United States of America; 4 Biology Department, Boston College, Chestnut Hill, Massachusetts, United States of America; 5 Institute for Technology Assessment, Department of Radiology, Massachusetts General Hospital, Boston, Massachusetts, United States of America; 6 Department of Neurosciences, University of California San Diego, La Jolla, California, United States of America; University of Nebraska, United States of America

## Abstract

**Background:**

Despite the advent of highly active anti-retroviral therapy (HAART), HIV-associated neurocognitive disorders continue to be a significant problem. In efforts to understand and alleviate neurocognitive deficits associated with HIV, we used an accelerated simian immunodeficiency virus (SIV) macaque model of NeuroAIDS to test whether minocycline is neuroprotective against lentiviral-induced neuronal injury.

**Methodology/Principal Findings:**

Eleven rhesus macaques were infected with SIV, depleted of CD8+ lymphocytes, and studied until eight weeks post inoculation (wpi). Seven animals received daily minocycline orally beginning at 4 wpi. Neuronal integrity was monitored *in vivo* by proton magnetic resonance spectroscopy and *post-mortem* by immunohistochemistry for synaptophysin (SYN), microtubule-associated protein 2 (MAP2), and neuronal counts. Astrogliosis and microglial activation were quantified by measuring glial fibrillary acidic protein (GFAP) and ionized calcium binding adaptor molecule 1 (IBA-1), respectively. SIV infection followed by CD8+ cell depletion induced a progressive decline in neuronal integrity evidenced by declining N-acetylaspartate/creatine (NAA/Cr), which was arrested with minocycline treatment. The recovery of this ratio was due to increases in NAA, indicating neuronal recovery, and decreases in Cr, likely reflecting downregulation of glial cell activation. SYN, MAP2, and neuronal counts were found to be higher in minocycline-treated animals compared to untreated animals while GFAP and IBA-1 expression were decreased compared to controls. CSF and plasma viral loads were lower in MN-treated animals.

**Conclusions/Significance:**

In conclusion, oral minocycline alleviates neuronal damage induced by the AIDS virus.

## Introduction

Early in the AIDS epidemic, severe neurological disorders including dementia were found to be caused by HIV [1], [2]. The use of antiretroviral drugs has greatly reduced the incidence of dementia, but neurocognitive complications of the virus, known as HIV-associated neurocognitive disorders (HAND), continue to be an important problem [3], [4], [5]. The general consensus of neuropathogenesis is that HIV enters the CNS primarily through the trafficking of virally infected/activated monocytes [6], [7]. This influx of infected/activated monocytes induces astrocytosis and microgliois. The production of neurotoxic substances by activated glia and infected macrophages as well as the presence of viral products likely cause neuronal cell injury and death [8], [9], which contribute to the neurologic impairment in HAND. Furthermore, the central nervous system can act as a reservoir for HIV due to the limited penetration of antiretroviral agents into the CNS [10].

The persistence of cumulative neurological disease despite antiretroviral therapy has led to a search for adjunctive therapies. Minocycline, an antibiotic with demonstrated neuroprotective properties, is being tested clinically in neuroAIDS (Clinial trial NCT00361257). The benefits of using minocycline are multifaceted including effects against apoptotic cell death, inflammation, microglial activation [11]–[14] and viral production [15], [16]. However, the benefits of MN and its mechanism of action in HAND remain unclear.

In this study, we conducted serial neuroimaging using *in vivo* ^1^H magnetic resonance spectroscopy (^1^H MRS) to assess the effects of minocycline on the brain injury produced by lentiviral infection in an accelerated macaque model of neuroAIDS. The rapidly progressing simian immunodeficiency virus (SIV)-infected macaque model uses a monoclonal antibody to persistently deplete the animal of CD8+ lymphocytes [17], [18] resulting in AIDS generally within 3 months with consistent development of astrogliosis, microgliosis, and neuronal injury [19]. Moreover, the alterations in brain metabolite concentrations in this model measured by *in vivo* ^1^H MRS, particularly N-acetylaspartate/Creatine (NAA/Cr), are very similar to those found in people with HAND. We longitudinally examined a group of rhesus macaques infected with SIV and CD8+ cell depleted (n = 11), a subset of which received minocycline (n = 7). We demonstrate an arrest of declining NAA/Cr, indicative of restoration of neuronal metabolism, and decreases in astrogliosis, microglial activation, and neuronal loss with minocycline treatment in SIV-infected macaques.

## Results

### CD8 T lymphocyte Depletion and Cohort Designation

Animals treated with anti-CD8 depleting antibody were either persistently depleted before reemergence of peripheral CD8 cells (>28 days, n = 8) or short-term depleted (<21 days, n = 3, recovered to 31,000/µL or 6.5% of WBC). Four MN-treated animals were persistently depleted and three were short-term depleted. Four untreated infected animals were persistently depleted. All analyses were performed on these 3 groups of animals: 1) infected, untreated, persistently CD8-depleted animals, 2) infected, MN-treated, persistently CD8-depleted animals, and 3) infected, MN-treated, short-term CD8-depleted, infected animals.

### Plasma and CSF Viral Loads

**Figure 1:**
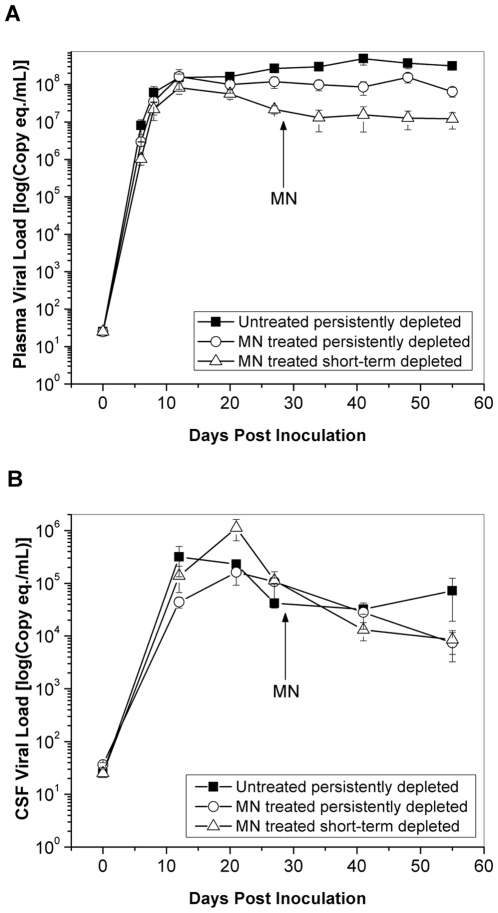
Viral loads in plasma and CSF. Mean viral load in untreated animals (solid squares), minocycline (MN)-treated persistently CD8-depleted animals (open circles) and MN-treated short-term CD8-depleted animals (open triangles) in plasma (A) and CSF (B) over the time course of SIV infection until 8 weeks post inoculation. Error bars represent standard error of the mean. The arrows represent the time point when minocycline (MN) treatment was initiated.

After SIV inoculation, the amount of virus in the peripheral blood plasma increased rapidly in all 3 cohorts, attaining levels greater than 10^7^ eq/mL by 12 days post inoculation (dpi) (p<0.001, Figure 1A). The plasma viral load in untreated animals continued to increase to a maximum of 4.9×10^8^ eq./mL, which was detected at both 41 dpi and 61 dpi. Increases in viral loads were statistically significant between 20 and 41 dpi in untreated animals (Holm's t-test p<0.001) while no further increase in plasma viral loads was observed in the MN-treated animals after 8 dpi. The animals whose CD8 T lymphocytes rebounded before 21 dpi and that received MN treatment had 1.5–2 orders of magnitude lower viral loads throughout the course of infection. The three cohorts had different plasma viral loads at 55 dpi (p = 0.003). Both minocycline cohorts had lower viral loads compared to untreated animals at 55 dpi (short-term CD8-depleted animals, p = 0.0017, and persistently CD8-depleted animals, p = 0.0034).

Figure 1B illustrates the CSF viral loads from the three cohorts over the duration of SIV infection. In all cohorts, peak levels of CSF virus were observed between 12 and 21 dpi. Significant declines from peak levels were only observed in the MN-treated animals. Persistently CD8-depleted, MN-treated animals showed a significant decline in CSF viral load between 21 dpi and the final scan (p = 0.002). Short-term CD8-depleted, MN-treated animals also had significant decreases from time of peak viral load and subsequent time points (21 dpi vs. 27 dpi p = 0.003, 21 dpi vs. 41 dpi p<0.001 and 21 dpi vs. 55 dpi p<0.001).

### Magnetic Resonance Spectroscopy

**Figure 2:**
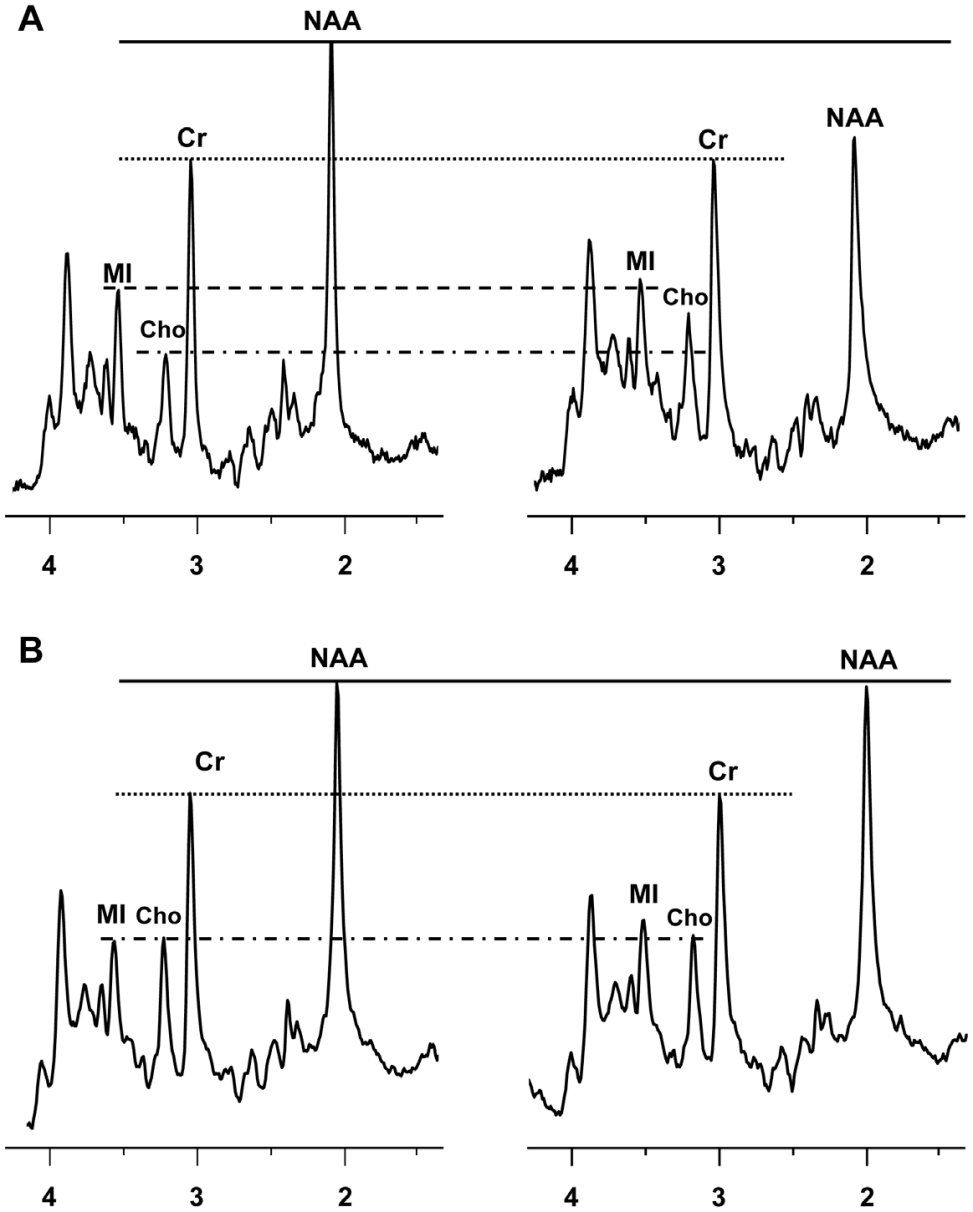
Comparison of ^1^H MR spectra of untreated and minocycline treated macaques. (**A**) ^1^H MR spectra from the white matter of a representative untreated macaque before infection and at 8 wpi. Spectra are scaled to the creatine (Cr) resonance. It can be seen from the spectra that NAA is decreased and Cho is increased at 8 wpi compared to baseline levels. (**B**) ^1^H MR spectra from the white matter of a representative minocycline treated (persistently CD8-depleted) macaque before infection and at 8 wpi. Again, the spectra are scaled to the creatine resonance. It is clear that NAA/Cr has not decreased as much and also that Cho/Cr has not increased at 8 wpi.

*In vivo* ^1^H MR spectra were acquired from the parietal cortex (PC), frontal cortex (FC), basal ganglia (BG) and white matter semiovale (WM). Figure 2 shows representative spectra acquired from an untreated animal prior to infection (Figure 2A, left) and at 8 weeks post inoculation (wpi) (Figure 2A, right) and from a minocycline treated, persistently CD8-depleted animal prior to infection (Figure 2B, left) and at 8 wpi (Figure 2B, right). ^1^H MR spectra acquired with short echo time (TE = 30 ms) are characterized by resonances primarily arising from N-acetylaspartate and N-acetylaspartylglutamate (collectively referred to as NAA), choline-containing compounds (referred to as Cho), *myo*-Inositol (MI), creatine-containing compounds (referred to as Cr) and the glutamate and glutamine concentrations (so-called Glx). Consistent with previous studies [19] the neuronal metabolism marker NAA is decreased while MI and Cho are increased in the untreated, CD8-depleted animals at 8 wpi compared to preinfection spectrum (Figure 2A). In contrast, the 8 wpi spectrum from the MN-treated animal is nearly the same as the preinfection spectrum except a modest increase in MI (Figure 2B). Uninfected, CD8-depleted controls exhibited no difference in pre- and post-depletion spectra (data not shown) [20]. N-acetylaspartate is a marker for neuronal integrity [21], [22] and creatine is a biomarker of energy metabolism likely localized to glial activation [23], [24]. Choline increases are indicative of membrane turnover due to glial activation and *myo*-Inositol elevation is considered to reflect increased glial cell activity [25].

#### Changes in NAA/Cr ratio

**Figure 3:**
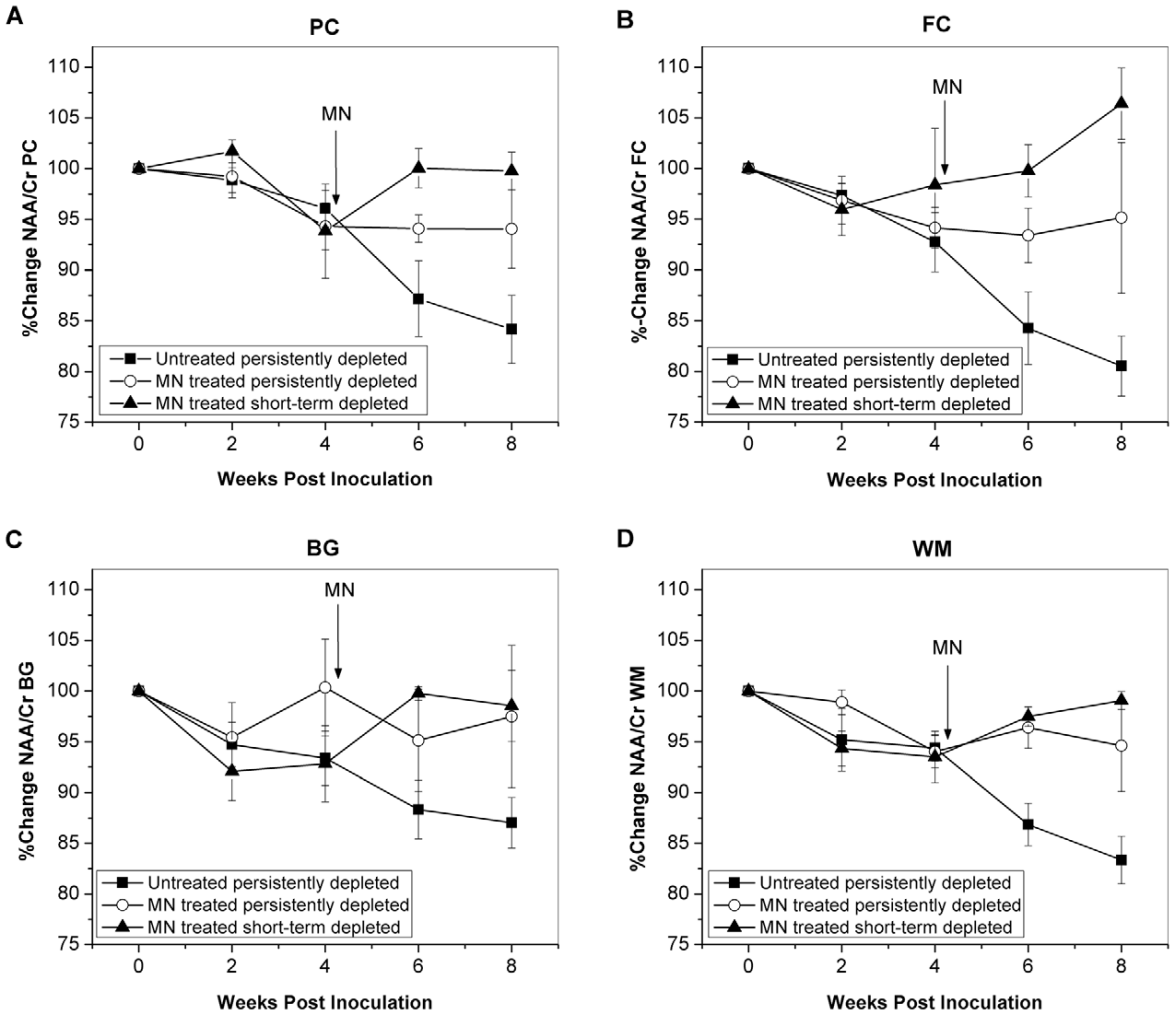
Recovery of neuronal marker NAA/Cr with minocycline treatment in SIV infection. NAA/Cr over the course of SIV infection in untreated animals (solid squares), minocycline (MN)-treated persistently CD8-depleted animals (open circles) and MN-treated short-term CD8-depleted animals (solid triangles). Mean NAA/Cr measured in the parietal cortex (**A**), frontal cortex (**B**), basal ganglia (**C**) and white matter semiovale (**D**) are plotted over time. Error bars represent standard error of the mean. The arrows represent the time point when minocycline (MN) treatment was initiated.

The mean percent changes in the levels of NAA/Cr as a measure of neuronal metabolism with respect to time after SIV infection in each brain region are graphically displayed in Figure 3. Analysis of the MR spectra from all eleven animals before treatment revealed decreases in NAA/Cr at 2 wpi in the BG (p = 0.009) and WM (p = 0.007), and at 4 wpi in the PC (p = 0.002), FC (p = 0.002) and WM (p<0.001). The four SIV-infected untreated animals (solid squares) had a 16% decrease in NAA/Cr in the PC over the 8 weeks following inoculation (p<0.001), as well as declines of 19% in the FC (p<0.001), 13% in the BG (p = 0.004) and 17% in the WM (p<0.001). The declines in NAA/Cr were significant at both 6 and 8 wpi when compared to pre-infection scans.

For the four SIV-infected, persistently CD8-depleted MN-treated animals (open circles), four weeks of MN treatment starting at 4 wpi resulted in a stabilization of NAA/Cr levels in all regions with partial recovery to values that were not significantly different from baseline. Treatment with MN resulted in progressive increase in NAA/Cr levels after treatment initiation in all brain regions in the three SIV-infected, short-term CD8-depleted, MN treated animals (solid triangles) (Figure 3).

**Figure 4:**
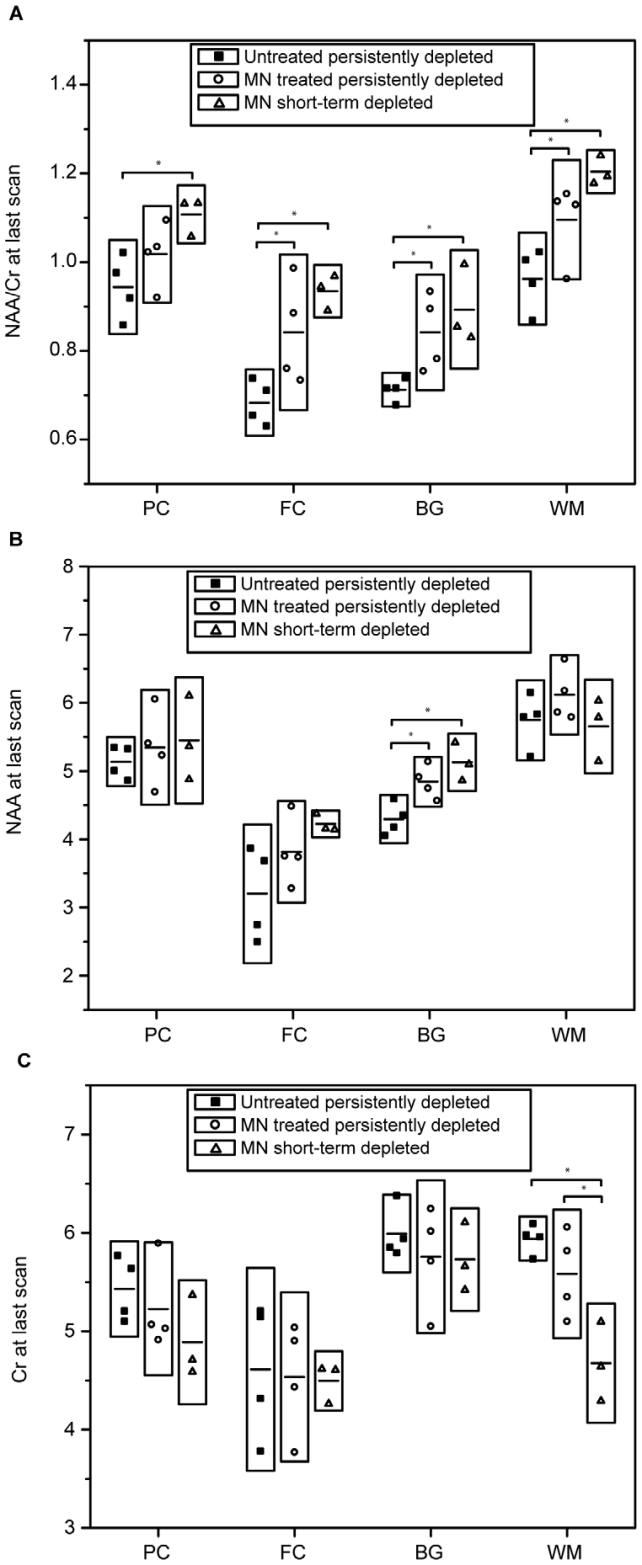
Minocycline prevents NAA declines and Cr elevation. (**A**) NAA/Cr at the last MR scan is significantly different among the three cohorts in all four brain regions by ANOVA (PC p = 0.034, FC p = 0.009, BG p = 0.024 and WM p = 0.0065). NAA/Cr was found to be significantly higher in MN-treated, persistently CD8-depleted animals compared to animals not treated with MN in the FC (p = 0.024), BG (p = 0.033) and WM (p = 0.029) by Least Square means t-tests. NAA/Cr was also significantly higher in all brain regions in MN treated, short-term CD8-depleted animals when compared to the untreated animals (PC p = 0.0057, FC p = 0.0035, BG p = 0.01, and WM p = 0.002). No significant differences between the two MN-treated cohorts were identified. (**B**) NAA concentrations at the last MR scan are higher in MN-treated animals than in untreated animals. NAA levels are significantly different among the three cohorts in the BG by ANOVA (p = 0.0062). NAA was found to be significantly higher in MN-treated, long-term CD8-depleted animals (p = 0.015) and MN treated, short-term CD8-depleted animals (p = 0.0024). No significant differences between the two MN-treated cohorts were identified. (**C**) Cr concentrations at the last MR scan are lower in MN-treated animals and are significantly different between each of the three cohorts in the WM by ANOVA (p = 0.0042). Cr was found to be significantly lower in MN-treated, short-term CD8-depleted animals (p = 0.0014) compared to untreated animals. Here, differences between the two MN treated cohorts were also identified (p = 0.0092) showing higher Cr level in long-term CD8-depleted animals. ***Horizontal bars within boxes represent mean values; height of each box corresponds to a factor of 1.5 times standard deviation.*** ** * indicates a significant difference (p≤0.05) between groups.

The SIV-infected untreated animals exhibited the lowest levels of NAA/Cr at the end of the study (8 wpi) in all brain regions examined (Figure 4). NAA/Cr was significantly higher in all brain regions in the MN-treated, short-term CD8-depleted animals when compared to the SIV-infected animals that did not receive MN animals. NAA/Cr was also significantly higher in the FC, BG and WM of the SIV-infected MN-treated, persistently CD8-depleted animals compared to untreated animals. Although NAA/Cr tended to be higher in those MN treated animals that experienced only short-term depletion of CD8 cells, the differences between the two MN-treated cohorts did not reach significance.

#### Changes in NAA and Creatine concentrations

The differences detected in the NAA/Cr ratio in various brain regions may be due to changes in either or both metabolites. In an attempt to identify whether changes in NAA and/or Cr are responsible for the changes observed in the ratio, NAA and Cr concentrations were estimated using tissue water as the internal standard. Due to the fact that there are over four orders of magnitude differences between the concentrations of the metabolites and water concentrations, the values have a much higher variance compared to the metabolite-metabolite ratios. Naturally, this makes obtaining statistical significance more difficult than for comparisons involving metabolite-metabolite ratios.

The mean NAA concentrations in untreated SIV-infected animals declined relative to baseline measurements in each brain region analyzed, but the differences were statistically significant only in the WM (PC -4% p = 0.68, FC -11% p = 0.068, BG -8% p = 0.16, WM -9% p = 0.03). The mean Cr concentrations in these untreated SIV-infected animals were elevated in all four regions, but statistical significance was found only in the parietal cortex and white matter (PC +14% p = 0.01, FC +11% p = 0.43, BG +6% p = 0.07 and WM +9% p = 0.01).

The initiation of minocycline treatment four weeks after inoculation in seven animals arrested the decline in NAA and increase in Cr concentrations in the brains of these animals. The effect of minocycline treatment and the combined effects of minocycline with partial immune reconstitution of the CD8 T cell population are best appreciated when the brain concentrations of NAA and Cr at the last data point are compared in the three cohorts. As shown in Figure 4B and C, by 8 wpi NAA concentrations were lower and Cr concentrations were higher in every brain region analyzed in the untreated animals when compared to MN-treated animals. Moreover, the greatest differences were between the untreated animals and MN-treated animals that experienced partial recovery of CD8 cells.

#### Changes in other metabolites

Significant changes in choline (Cho) were observed in all eleven animals during the first four weeks of SIV infection (p<0.001), with an increase at 2 wpi followed by a decrease to baseline values or below, as seen in the WM. In the four SIV-infected, persistently depleted, untreated animals Cho again increased above baseline levels at 8 wpi (PC +22%, FC +9%, BG 10%, WM 16%). The increases between 4 and 8 wpi were statistically significant in the PC (p = 0.003) and WM (p = 0.003) (data not shown).

**Figure 5:**
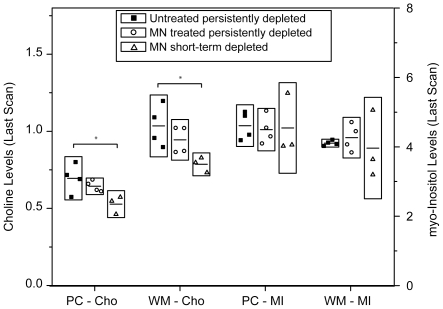
Minocycline reduces elevation of choline, not myo-Inositol. Choline concentrations (left) and myo-Inositol concentrations (right) in the PC and WM at the time of the last MRI scan comparing the three cohorts. Cho concentrations are significantly different among the three cohorts in the PC (p = 0.033) and WM (p = 0.035) by ANOVA. Cho levels are significantly lower in MN-treated, short-term CD8-depleted animals (PC p = 0.01 and WM p = 0.012) compared to untreated animals. Differences in MI concentrations at the last MR scan were not statistically significant among the three cohorts by ANOVA. ***Horizontal bars within boxes represent mean values; height of each box corresponds to a factor of 1.5 times standard deviation.*** ** * indicates a significant difference (p≤0.05) between groups.

In contrast, increases in Cho after 4 wpi were not significant in MN-treated, short-term CD8-depleted animals. In MN-treated, persistently CD8-depleted animals, increases in Cho after 4 wpi were not significant in the parietal cortex; however, Cho did increase significantly in the WM (p = 0.003) and in the BG (p = 0.001). Furthermore, Cho levels in the animals at their last scan before sacrifice was significantly different among the three cohorts in the PC and WM (Figure 5) with the lowest Cho levels observed in the MN-treated, short-term CD8-depleted animals.

*Myo*-Inositol (MI) levels show an increase at 2 and 4 weeks after inoculation and then normalization to baseline values (data not shown). Within the first 4 weeks of infection MI significantly increased at 2 weeks in the FC and WM (p<0.001 and p = 0.027, respectively) and remained elevated at 4 weeks for all eleven animals. At later time points, even without treatment, MI decreases and becomes indistinguishable from baseline levels (Figure 5). With MN treatment no significant differences in the MI or MI/Cr trends could be observed.

During the first four weeks of SIV infection, glutamate and glutamine concentrations (Glx) decreased in the parietal cortex at 2 and 4 wpi compared to baseline values (p = 0.002). The same trends are observed in the other brain regions; however, changes did not reach statistical significance. After 4 weeks Glx levels did not change significantly. The Glx levels of the three cohorts at the animals' last scan before sacrifice were not significantly different from each other. Due to the large experimental error and variance in the measurements of glutamine and glutamate, larger cohort sizes may be needed to identify any statistically significant changes.

### Neuropathology

#### Experimental information and histopathology

Table 1 summarizes the experimental information and histopathological findings for the brains of the animals included in this study. SIV-infected, persistently depleted, untreated animals euthanized at 8 wpi (n = 4) exhibited infiltrates, gliosis, cortical neuronal degeneration, and satellitosis. One animal had occasional multinucelated giant cells, the classical hallmark of SIV encephalitis (SIVE). Additionally, two of the SIV-infected, untreated animals developed occasional accumulations of perivascular macrophages consistent with mild SIVE. In total, three of the four (75%) untreated animals developed infiltrates in the brain within 8 wpi. In contrast, one of four persistently depleted MN-treated (25%) and two of three short-term depleted MN-treated (66%) animals had identifiable infiltrates in the brain at 8 wpi. Yet, all MN treated animals still exhibited signs of mild to moderate gliosis. Most MN-treated animals had mild to moderate neurodegeneration and satellitosis while untreated animals had moderate to severe neurodegeneration and satellitosis.

**Table 1:** Summary of clinical information and CNS histopathology based on H&E.

Animal	CD8 T Cell Depletion	Treatment	Survival (days)	AIDS	Encephalitis (infiltrates)	Gliosis	Satellitosis	Neurodegeneration
**M5207**	Persistent	None	57	SIV-related	Y (mild)	moderate	severe	severe
**M5407**	Persistent	None	57	Y	Y (mild)	moderate	moderate	moderate
**M7207**	Persistent	None	62	Y	N	mild	moderate	moderate
**M1308**	Persistent	None	62	Y	Y (moderate *)	mild	severe	severe
**M7307**	Persistent	MN	62	SIV-related	Y (moderate)	moderate	moderate	moderate
**M1508**	Persistent	MN	60	N	N	mild	moderate	moderate
**M1608**	Persistent	MN	60	N	N	mild	mild	mild
**M3408**	Persistent	MN	55	Y	N	mild	moderate	moderate
**M7407**	Partial Recovery	MN	61	SIV-related	Y (mild)	mild	mild	mild
**M7507**	Partial Recovery	MN	62	SIV-related	N	mild	moderate	moderate
**M1408**	Partial Recovery	MN	62	N	Y (mild)	moderate	moderate	moderate

MNGCs: multinucleated giant cells. * rare MNGCs.

Objective scoring: Five regions within the H&E section of frontal cortex were examined and scored at 10× magnification using the following criteria for encephalitis, satellitosis, and neurodegeneration. Gliosis scoring was performed on 5–8 H&E brain sections.

Encephalitis scoring: Mild- perivascular infiltrated in one brain region; Moderate- perivascular infiltrates in two or more regions plus rare MNGCs; Severe-perivascular infiltrates in all regions and frequent MNGCs.

Gliosis scoring: Mild- increased cellularity in white matter in one brain region; Moderate- increased cellularity in white matter in two or three regions plus occasional microglial nodules; Severe- increased cellularity in white matter in all regions with frequent microglial nodules.

Satellitosis scoring: Neurons surrounded by 3 or more glial cells are characterized as mild (occasionally occurring); moderate (frequently) and severe (numerous, in addition to clusters of 3 or more glial cells at sites of neuronal drop-out).

Neurodegeneration scoring: Mild- occasional angular, shrunken neurons; Moderate- frequent angular, shrunken neurons; Severe- numerous angular, shrunken neurons and neuronal drop-out.

#### Synaptophysin, Microtubule-Associated Protein 2 and neuronal counts

For all following *post-mortem* studies a control cohort of four age-matched uninfected, CD8 T-lymphocyte depleted animals was introduced to reveal changes from “baseline”. The uninfected CD8-depleted cohort was chosen to detect the effect of minocycline on SIV infection and not on CD8 depletion. Using *in vivo* MR spectroscopy and *post-mortem* IHC, our group has previously shown that CD8 depletion without SIV infection does not change brain metabolism or provoke neuronal or glial degradation [20].

**Figure 6:**
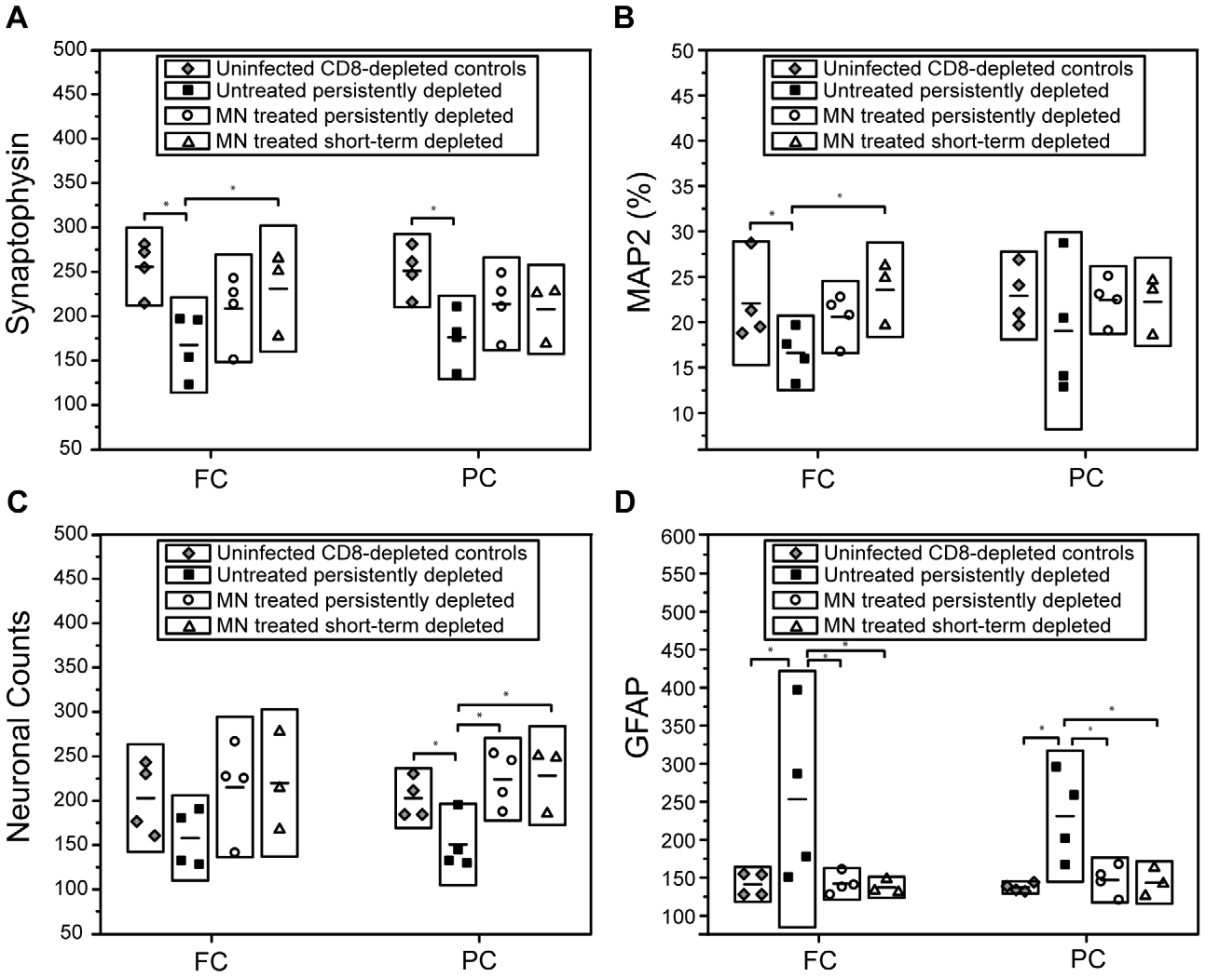
Minocycline preserves neuronal integrity and decreases astrogliosis. Synaptophysin (SYN), Microtubule-associated protein 2 (MAP2), neuronal counts, and glial fibrillary acetic protein (GFAP) levels were quantified in the frontal and parietal cortices of 1) four uninfected CD8-depleted control animals, 2) four SIV-infected, persistently CD8-depleted untreated animals, 3) four MN-treated, SIV-infected, persistently CD8-depleted animals and 4) three MN-treated, SIV-infected, short-term CD8-depleted animals. Horizontal bars within boxes represent mean values; height of each box corresponds to a factor of 1.5 times standard deviation. * indicates a significant difference (p≤0.05) between groups. (**A**) Frontal cortex SYN levels were decreased in SIV-infected CD8-depleted untreated animals versus uninfected CD8-depleted controls (p = 0.007). Synaptophysin was higher in MN-treated short-term depleted animals compared to untreated animals (p = 0.05). No statistical differences between controls and either of the two MN-treated cohorts were observed. Parietal cortex SYN levels were also decreased in SIV-infected CD8-depleted untreated animals versus controls (p = 0.006). (**B**) MAP2 levels were decreased in SIV-infected CD8-depleted untreated animals versus controls (p = 0.046) and versus MN-treated, short-term CD8-depleted animals (p = 0.02). Parietal cortex MAP2 levels were higher in controls and MN-treated animals compared to untreated animals; however, these differences fail to yield statistical significance. (**C**) In the parietal cortex SIV-infected untreated animals had lower neuronal counts compared to controls (p = 0.031). There were decreased numbers of neurons in untreated animals compared to both the MN-treated persistently CD8-depleted animals (p = 0.005) and short-term CD8-depleted animals (p = 0.006). (**D**) Frontal cortex GFAP levels were increased in the SIV-infected, CD8-depleted untreated cohort compared to uninfected CD8-depleted controls (p = 0.023). GFAP levels in both MN-treated persistently CD8-depleted animals (p = 0.023) and short-term CD8-depleted animals (p = 0.027) were significantly decreased compared to the untreated cohort. Increases in parietal cortex GFAP levels were identified in the SIV-infected, CD8-depleted untreated cohort compared to uninfected controls (p = 0.002). GFAP levels in both MN-treated persistently CD8-depleted animals (p = 0.0041) and short-term CD8-depleted animals (p = 0.0026) were significantly decreased compared to the untreated cohort.

Decreases in the expression of synaptophysin (SYN), an integral protein in presynaptic terminals, and microtubule-associated protein 2 (MAP2), a marker for neuronal cell bodies and dendrites, reflect neurodegeneration severity and have been found to correlate to the degree of neurocognitive impairment in persons with HIV [26]. These neuronal markers were measured using immunohistochemistry as well as neuronal counts, and the results are displayed in Figure 6 (A, B and C). All three measures of neuronal integrity were lower in the frontal and parietal cortices in SIV-infected, CD8+ cell-depleted animals when compared to uninfected CD8+ cell-depleted control animals. Measurements of these markers in the frontal and parietal lobes of macaques treated with oral MN starting at 4 wpi revealed levels of SYN, MAP2 and neuronal counts that were not significantly different from uninfected CD8-depleted controls.

There was a statistically significant difference in frontal cortex SYN levels among the four cohorts (ANOVA p = 0.04, Figure 6A). Synaptophysin was higher in MN-treated short-term depleted animals compared to the untreated cohort. Furthermore, parietal cortex SYN levels were significantly different among the four cohorts (ANOVA p = 0.04). In the PC SYN levels were higher in MN-treated persistently depleted animals compared to the untreated animals, but the difference did not reach significance.

MAP2 levels showed a trend towards significance in the frontal cortex among the four cohorts (ANOVA p = 0.09, Figure 6B) with increased MAP2 levels in MN-treated, short-term depleted animals compared untreated animals. Parietal cortex MAP2 levels were higher in controls and MN-treated animals compared to untreated animals; however, these differences failed to reach statistical significance (ANOVA p = 0.63).

While a higher number of neurons was observed in the frontal cortex of uninfected animals and MN-treated animals compared to the untreated cohort, these differences were not statistically significant (ANOVA p = 0.27). There was, however, a statistically significant difference in parietal cortex neuronal counts among the four cohorts (ANOVA p = 0.017) showing decreased numbers of neurons in untreated animals compared to both the MN-treated persistently CD8-depleted animals and short-term CD8-depleted animals.

#### GFAP – marker of astrogliosis

Glial fibrillary acidic protein (GFAP) is the principal intermediate filament in mature astrocytes. Astrocytes rapidly synthesize GFAP in response to a neurologic insult, and it is the most commonly used marker of astrogliosis. GFAP levels were quantified in the frontal and parietal cortex of the same four cohorts of animals used for the neuronal marker determinations described in the previous section (Figure 6D, ANOVA p = 0.05 and ANOVA p = 0.0062, respectively). GFAP was significantly elevated in the frontal and parietal cortices of the SIV-infected, CD8+ cell-depleted animals when compared to the uninfected, CD8-depleted control animals and the two cohorts that were treated with MN. No statistical difference between the uninfected, CD8-depleted controls and either of the two MN-treated cohorts was observed.

#### IBA-1 – marker of microglial activation

**Figure 7:**
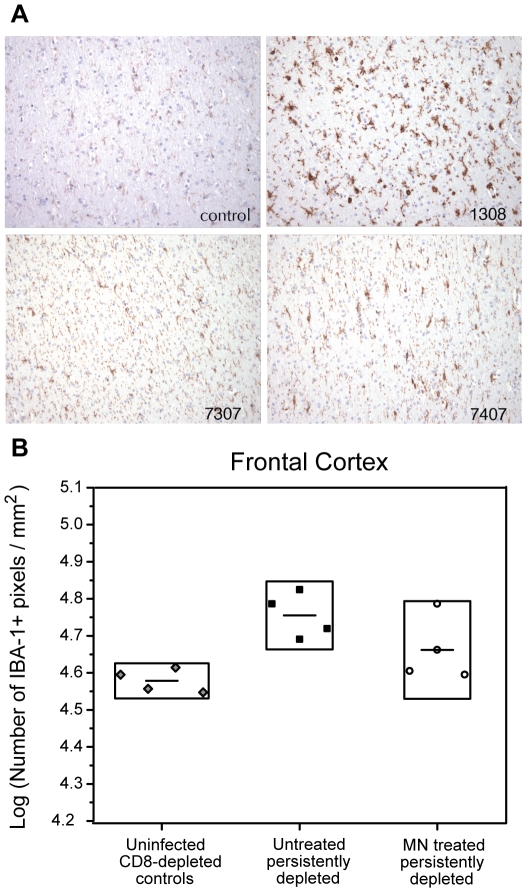
Minocycline reduces microglial activation, assessed by ionized calcium binding adaptor molecule 1 (IBA-1) expression. (**A**) IBA-1 expression in four representative sections from each of an uninfected, CD8-depleted animal (**control**), an untreated SIV-infected CD8-depleted animal (**1308**), a MN-treated persistently CD8-depleted animal (**7307**) and a MN-treated short-term CD8-depleted animal (7407). Visual inspection reveals less IBA-1 expression in the MN-treated animals. (**B**) Immunohistochemistry image analysis for IBA-1 in brain tissue sections of the frontal cortex. Data is given for four uninfected, CD8-depleted animals which served as controls (diamonds), four untreated SIV-infected CD8-depleted animals sacrificed at 8 wpi (solid squares) and four minocycline (MN)-treated (persistently CD8-depleted) animals. The mean for each cohort is given as a horizontal black line within its respective box. Upper and lower boundaries of the boxes correspond to a factor of 1.5 times the standard deviation. There was a statistically significant difference in frontal cortex IBA-1 levels among the three cohorts (p = 0.012). There are more IBA-1 positive particles/mm^2^ in the SIV-infected, CD8-depleted, untreated cohort compared to -uninfected, CD8-depleted controls (p = 0.004). There was no statistically significant difference in IBA-1 positive particles/mm^2^ in MN-treated animals compared to the uninfected CD8-depleted controls.

Calcium binding adaptor protein 1, IBA-1 is expressed by resting microglia and is upregulated when these cells are activated [27]. Widespread microglial activation accompanied by intense staining of IBA-1 was observed at 8 wpi in brains of the animals that were persistently CD8-depleted (Figure 7A) compared to uninfected, CD8-depleted control animals. There was substantially less IBA-1 expression in brains of animals from both MN-treated cohorts indicating reduced microglial activation.

To quantify the degree of IBA-1 upregulation induced by SIV infection in CD8-depleted animals, immunohistochemistry computer image analysis was performed on brain sections from uninfected, CD8-depleted control animals along with those from the two SIV-infected, persistently CD8-depleted cohorts, of which included one cohort that was MN-treated and one untreated cohort. We did not perform computer-aided image analyses on IBA-1 for the three MN-treated, short-term depleted animals and we recognize that this was an experimental flaw in our study. Figure 7B shows the IHC image analysis for IBA-1 of brain tissue sections from the frontal cortex of 1) the uninfected, CD8-depleted control cohort, 2) the SIV-infected, untreated, CD8-depleted cohort and 3) the four SIV-infected, MN-treated, persistently CD8-depleted animals. There was a statistically significant difference in frontal cortex IBA-1 levels among the three cohorts (p = 0.012). There were more IBA-1 positive particles/mm^2^ in the SIV-infected, untreated, CD8-depleted cohorts compared to the uninfected, CD8-depleted controls (p = 0.004) while there was no statistically significant difference in IBA-1 positive particles/mm^2^ in MN-treated animals compared to the controls. In the parietal cortex, no significant differences in IBA-1 levels among the cohorts were observed.

## Discussion

HIV-associated neurocognitive disorders continue to be a significant problem despite the use of highly active anti-retroviral drugs (HAART), and this has motivated a search for adjunctive therapies. The SIV-infected accelerated macaque model of neuroAIDS in combination with *in vivo* ^1^H MR spectroscopy provides an exceptional opportunity to efficiently explore drug therapies that can control neuronal injury [19]. Using this model oral minocycline was found to be neuroprotective, detected by stable levels of N-acetylaspartate, and to reverse increased high energy metabolism reflected by increased Cr, most likely related to glial activation. Clues into the mechanism(s) responsible for neuroprotection include the reversal of astrogliosis, reduction of microglial activation, and reductions of CSF and plasma viral loads. HIV-related neurological disease appears to be initiated by the trafficking of infected monoctyes into the brain [28], [29]. Once in the brain, infected macrophages and microglia initiate a cascade of events that include release of viral proteins, cytokines and chemokines [30], [31]. This is accompanied by significant astroglial and microglial activation [32], [33]. With disease progression, there is continued influx of inflammatory cells and ultimately the formation of productive multinucleated giant cells. During these events, several neurotoxic substances are released leading to neuronal injury and death, together which characterize the biological substrate of the neurocognitive disorders in question.

The SIV-infected rhesus macaque shares very similar pathology with HIV-infected human patients, including the development of AIDS, disease of the CNS, and cognitive or behavioral deficits [34]–[38]. However, because of its parallels with HIV pathogenesis, the traditional SIV macaque model is hindered by the low rate of development of SIVE along with the relatively long latent period of the virus. In addition, only 25–30 percent of infected macaques develop encephalitis [34], [39]. These factors conspire to make the testing of potential drug therapies extremely challenging. Thus, attention has focused on two rapidly progressing SIV/macaque models. One employs pig-tailed macaques (Macaca nemestrina) that are co-inoculated with immunosuppressive virus (SIV/DeltaB670) and a neurovirulent molecularly cloned virus (SIV/17E-Fr) [40]–[43]. The second model retains the use of the SIV-infected rhesus macaque, but uses a monoclonal antibody to deplete the animal of CD8+ lymphocytes to accelerate CNS disease progression [17]–[19], [44], [45]. While the development of SIV-induced neuronal injury is much more rapid in the accelerated CD8 depletion model of neuroAIDS compared to that in humans, the underlying biology leading to neuronal injury appears similar. The hallmark neuropathological abnormalities observed in humans are also present in this model including the accumulation of viral-laden perivascular macrophages, encephalitis, astrogliosis, microgliosis, and neuronal injury.

Moreover, the neuroimaging abnormalities observed in this animal model are similar to changes in HIV-infected patients that correlate with neurocognitive impairment. Chang *et al*. reported 10% decreases in NAA/Cr in patients with mild cognitive motor dysfunction [46], while 15–28% decreases in NAA or NAA/Cr have been reported for those with severe cognitive impairment or AIDS Dementia Complex [47]–[50].

Declines in the neuronal marker NAA and in NAA/Cr have been found to correlate with disease severity of macaque SIV encephalitis [51]–[54]. Increased Cr levels have been reported in antiretroviral-naïve, HIV-infected patients [55], [56] as well as in macaques shortly after SIV infection [57]. The increase in Cr most likely reflects increased metabolic demands of activated glia and inflammatory cells [23], [24]. Other changes include elevations in Cho [58], [59], [60], which likely reflect increased membrane turnover occurring during glial activation and inflammation [50], [52], [61], [62]. Similar metabolic changes were observed in the present study, and those changes were reversed or arrested by treatment with minocycline.

Minocycline treatment beginning at 4 wpi was found to arrest further decrease in NAA/Cr in SIV-infected, persistently CD8 T cell depleted animals. In addition, a complete recovery of NAA/Cr was observed in MN-treated animals that had partial immune reconstitution of the CD8 T cell population. The recovery of the NAA/Cr ratio was found to be due to increases in NAA, suggesting neuronal recovery, and decreases in Cr, possibly reflecting downregulation of glial and inflammatory cell activation. These salutary effects observed by brain imaging were accompanied by clinical improvement including weight gain (data not shown).

The beneficial effects of MN on neuronal health were confirmed by *post-mortem* analyses. Synaptophysin (SYN) is a membrane-bound glycoprotein of the small synaptic vesicles, consistently expressed in all pre-synaptic nerve endings of the CNS [63]. MAP2 is a high-molecular-weight protein that localizes to the dendritic compartment of neurons and is involved in microtubule assembly. Both SYN and MAP2 have been shown to be very useful in measuring the degree neuronal damage occurring due to neuroAIDS [64]. Untreated SIV-infected, CD8+ cell-depleted animals demonstrated markedly decreased expression of SYN and MAP2 compared with uninfected controls, while MN-treated animals did not differ from uninfected controls. Furthermore, minocycline treatment resulted in increased neuronal counts compared to untreated SIV-infected animals.

Minocycline has been found to be neuroprotective in models of several neurological diseases including ischemia [65], [66], multiple sclerosis [67], Parkinson's disease [68], Huntington's disease [69] and recently in neuroAIDS [15], [16], [70], [71]. The neuroprotective properties of MN are thought to be mediated at least partly through downregulation of microglial activation [11], [72], [73]. The possible role of this mechanism in the present study is supported by the finding that MN-treated animals had lower levels of IBA-1 expression, a marker increased with microglial activation, compared to the untreated cohort. Levels of IBA-1 expression in the MN-treated animals, however, still remained higher than levels in uninfected animals. Additionally, GFAP was decreased in MN-treated animals compared to untreated SIV-infected macaques. The possibility that four weeks of MN treatment does not fully reverse glial activation was also supported by the observation of persistent mild gliosis in treated animals by hematoxylin and eosin staining. These results suggest that while short-term MN treatment arrests neuronal injury, longer treatments of MN may be necessary to fully normalize glial activity.

Another possible mechanism for neuroprotection is suggested by the finding of lower viral loads in MN-treated, SIV-infected macaques. Indeed, complete recovery of NAA/Cr was observed in the animals with partial immune reconstitution of the CD8 T cell population. These animals had the lowest levels of plasma viral loads of the three SIV-infected cohorts. A similar effect by MN in a different nonhuman primate model of accelerated AIDS was reported by Zink [16]. Lower plasma viral loads could lead to reduced trafficking of infected cells into the CNS, and this alone may be sufficient to allow the innate recovery mechanisms in the brain to overcome continuing effects of residual virus. Evidence supporting this approach includes the observation of neuronal injury reversal in this model, which occurred when macaques were treated with combination anti-retroviral drugs that did not penetrate the CNS [19]. The mechanism by which minocycline is able to reduce viral loads has yet to be elucidated. One possibility is that MN affects immune cells outside the CNS in a manner similar to microglia in the brain. That is, it may downregulate immune cells that normally are productively infected by the virus. Si et al. [15] and Zink et al. [16], [70] have demonstrated that the anti-viral effect of MN reduces viral burden in microglial cultures and in the brain, respectively. It has been suggested that the drug's anti-viral activity is mediated by its binding to the HIV integrase activity site [71].

All four untreated and four of the seven minocycline-treated animals had persistent depletion of CD8+ lymphocytes. However, three of the treated animals had partial recovery of CD8+ lymphocytes. This latter cohort was distinct from the other two with respect to viral loads, imaging, neuropathology and other measures. The better profile of this cohort demonstrates the essential role of CD8+ lymphocyte depletion in producing the neuroAIDS spectrum up to SIV encephalitis. It also demonstrates that while MN treatment alone is insufficient to fully protect the brain in this model and, presumably, the brain in HIV-infected individuals, a combination of approaches may lead to superior results. Thus, the best strategy to treat neuroAIDS may be with combination therapy of minocycline and antiretroviral therapy.

Some studies have found conflicting results with respect to the neuroprotective properties of minocycline [73]–[77]. In fact, some have even reported a harmful effect on patients with amyotrophic lateral sclerosis [78]. Therefore, it is essential to determine minocycline's mechanism of action in preparation for further clinical trials for neuroAIDS treatment. Furthermore, not all subjects may respond equally to MN treatment, thus, more studies to explore genetic or other confounding factors are necessary to understand differences in therapeutic effects.

In conclusion, short-term administration of oral minocycline resulted in significant neuroprotection in the SIV-infected macaque accelerated model of neuroAIDS. The study also demonstrated the efficacy of *in vivo* ^1^H MR spectroscopy to noninvasively assess minocycline as a neuroprotective agent in neuroAIDS and suggests that the integration of MR spectroscopy should be considered for use in clinical trials of minocycline in patients with HAND.

## Materials and Methods

### Ethics Statement

All animal studies were performed in accordance with federal laws and regulations, international accreditation standards, and institutional policies, including approval by the Massachusetts General Hospital Subcommittee on Research Animal Care and the Institutional Animal Care and Use Committee of Harvard University. All animals received environmental enrichment and were monitored daily for evidence of disease and changes in attitude, appetite, or behavior suggestive of illness. Appropriate clinical support was administered under the direction of the attending veterinarian and included analgesics, antibiotics, intravenous fluids, and other supportive care. Animals were euthanized when they presented with advanced stages of AIDS; criteria for euthanasia included 15% weight loss in two weeks, unresponsive opportunistic infection, persistent anorexia, intractable diarrhea, progressive neurologic signs, significant cardiac or pulmonary signs or other serious illness.

### Non-human Primates

Eleven 4–5 years old (7 male) rhesus macaques (Macaca mulatta) were included in this study (Table 1). All animals were inoculated with SIVmac251 virus (10 ng SIVp27, i.v.) and their CD8+ T-lymphocytes were depleted with antibody targeted against CD8 (cM-T807) at 6, 8 and 12 days post inoculation (dpi). In this prospective study all infected animals were studied until 8 weeks post inoculation before being euthanized. Seven animals were treated orally with 4 mg/kg/day minocycline (2 mg/kg twice a day) starting at 4 wpi. Minocycline powder was compounded by Birds' Hill Compounding Pharmacy (Needham, MA) into cherry flavored syrup, which was further diluted into sports drinks or juices. The appropriate dose was given via a syringe to ensure animals consumed the entire doses. All animals selected for treatment showed high acceptance levels. No adverse effects or side effects were observed.

Animals were scanned two times before infection and biweekly until sacrifice. For MR imaging, each animal was tranquilized with 15–20 mg/kg intramuscular ketamine hydrochloride and intubated to ensure a patent airway during the experiment. Intravenous injection of 0.4 mg/kg atropine was administered to prevent bradycardia. Continuous infusion of approximately 0.25 mg/kg/min propofol was maintained throughout imaging via catheter in a saphenous vein. Heart rate, oxygen saturation, end-tidal CO_2_ and respiratory rate were monitored continuously. A heated water blanket was used to prevent hypothermia. All animals were anesthetized with ketamine-HCl and euthanized by intravenous pentobarbital overdose.

One additional cohort of four uninfected CD8-depleted macaques (4.6 years old, 4 males) were included for the *post-mortem* evaluations as control cohort.

### MRI and MRS

All experiments were performed with a 3 Tesla whole-body imager (Magnetom TIM Trio, Siemens AG, Erlangen, Germany) using a circularly polarized transmit-receive extremity coil. First, a three-plane localizer was performed to position the monkey in the coil. In this manner, voxel placement was highly reproducible. To image-guide the ^1^H MRS volume of interest (VOI), sagittal and axial turbo spin echo MRI were acquired at 140×140 mm^2^ field of view (FOV), TE = 16 ms and 512×512 matrix size. Other imaging parameters include a 2 mm slice thickness for sagittal images, a 1.2 mm slice thickness for axial images and repetition times (TR) of 4500 ms and 7430 ms for sagittal and axial images, respectively.

Single voxel ^1^H MR spectroscopy was performed in the parietal cortex (PC), frontal cortex (FC), basal ganglia (BG) and white matter semiovale (WM) using a point resolved spectroscopy sequence (PRESS) with water suppression enhanced through T_1_ effects (WET) [79] using the following parameters: TE = 30 ms, TR = 2500 ms and 192 acquisitions.

All spectra were processed offline using the LCModel software package [80] to determine the quantities of the brain metabolites N-acetylaspartate and N-acetylaspartylglutamate (collectively referred to as NAA), choline-containing compounds (referred to as Cho), myo-inositol (MI), and creatine-containing compounds (referred to as Cr). Metabolite ratios were calculated with respect to creatine. Furthermore, metabolite concentrations were estimated using the unsuppressed water signal from the same voxel, which served as the internal standard resulting in institutional units reflecting millimolar concentration.

### Viral Loads and Flow Cytometry

Blood and CSF were drawn before every MR scan, blood was centrifuged, and plasma and CSF were stored at −80°C until study endpoint. Virion-associated SIV RNA in plasma was measured by using a real-time reverse transcription-PCR assay on an Applied Biosystems (Foster City, CA) Prism 7700 sequence detection system with a threshold sensitivity of 100 copy eq/mL, as previously described [81]. Results represent mean values of duplicate determinations.

CD8+ T lymphocyte depletion was monitored by flow cytometry prior to infection and CD8 depletion and weekly thereafter. Flow cytometric analyses were performed with 100-µl aliquots of blood incubated with fluorochrome-conjugated antibodies including anti–CD3-APC (clone FN18; BioSource International), anti–CD4-FITC (OKT4; Ortho Diagnostic Systems), anti–CD8-PE (DK25; DakoCytomation), and anti–CD20–PE–Texas Red (B1; Beckman Coulter). Following antibody incubation for 15 minutes at room temperature, cells were washed twice with PBS containing 2% FBS; erythrocytes were lysed using ImmunoPrep Reagent System (Beckman Coulter); and samples were washed with PBS, resuspended in 2% formaldehyde in PBS and analyzed on a FACSCalibur flow cytometer (BD). The absolute number of CD8+ T lymphocytes was determined by multiplying the percentage of CD8+ CD3+ T cells by absolute lymphocyte counts obtained using a standard veterinary 3-point WBC differential, CBC Hematology Analyzer (Hema-True, HESKA).

### Histology and Immunohistochemistry

At day of sacrifice, all animals were anesthetized with ketamine-HCl and euthanized by intravenous pentobarbital overdose. Animals were perfused with 4 liters of chilled saline, and CNS tissues were collected in 10% neutral buffered formalin, embedded in paraffin, sectioned at 6 µm and stained with hematoxylin and eosin (H&E). All H&E sections were evaluated by a neuropathologist.

Microglial activation was assessed by quantifying calcium binding adaptor protein 1 (IBA-1). Frontal and parietal cortical brain sections of the four untreated animals euthanized at 8 wpi and the four persistently CD8-depleted animals were incubated with rabbit anti-IBA-1 (Wako Corp. Japan) to determine the amount of microgliosis. In addition, frontal cortex and parietal cortex from four uninfected, CD8-depleted animals served as the control cohort for the IBA-1 analyses. Images of tissue sections were captured without manipulation using an Olympus 3-CCD T60C color video camera mounted on an Olypus Vanox-SI microscope and analyzed using NIH Image J software.

Quantitative immunohistochemistry for glial fibrillary acidic protein (GFAP), synaptophysin (SYN), microtubule-associated protein 2 (MAP2) and neuronal counts was performed on fifteen animals: 1) four uninfected CD8-depleted control animals, 2) the four SIV-infected, CD8-depleted untreated animals sacrificed at 8 wpi, 3) the four MN-treated, persistently CD8-depleted SIV-infected animals and 4) the three MN-treated, short-term CD8-depleted, SIV-infected animals.

The degree of reactive astrogliosis was assessed with the monoclonal anti-glial fibrillary acidic protein (1∶1000; Boehringer Mannheim, Indianapolis, IN). 5 µm-thick paraffin sections from the frontal cortex were immunolabeled overnight with these monoclonal antibodies followed by biotinylated horse anti-mouse immunoglobulin G, avidin-horseradish peroxidase (Vectastain Elite kit; Vector, Burlingame, CA), and reacted with diaminobenzidine tetrahydrochloride and peroxide (0.03%). The integrity of the synapses was evaluated with the monoclonal antibody against synaptophysin (1∶10) (Boehringer Mannheim, Indianapolis, Ind). The status of neuronal dendrites was evaluated by using monoclonal antibody against microtubule-associated protein 2 (MAP2) (Boehringer Mannheim).

Levels of GFAP, synaptophysin, and MAP2 were estimated by means of computer-aided image analysis, as previously described [82]. Immunoreactivity was semiquantitatively assessed as corrected optical density by using a microdensitometer (Quantimet 570C; Leica, Microsystem Cambridge, UK). For this purpose, three immunolabeled sections were analyzed from each case. As previously described [53], [61], [82], the system was first calibrated with a set of filters of various densities, and 10 images were obtained for each section at ×100 magnification. After the area of interest (layers 2–5) was delineated with the cursor, the optical density within that area was obtained. The optical density in each image was averaged and expressed as the mean per case. The units of all measurements for GFAP and synaptophysin are in arbitrary optical density units and range from 0 to 500 (i.e., 0 indicates all light is allowed to pass through the sample, while 500 indicates no light is allowed to pass through the sample). All values are expressed as mean ± standard error of the mean. MAP2 is given in percent area of the neuropil covered by MAP2 immunoreactive dendrites.

The number of cortical neurons was quantified by using stereologic evaluation. Formalin-fixed paraffin sections (7 µm thick) from the frontal and parietal cortex were stained with cresyl violet for subsequent computer-aided image analysis as previously described [83]. The volume-weighted neuronal number density was calculated by dividing neuronal volume fraction by the mean number-weighted neuronal volume. These estimations were derived by using an oil immersion objective lens (numerical aperture, 1.25) at 100× magnification to analyze contiguous fields of 65×65 µm, with four such fields across and down through full thickness of cortex. Neurons were included only if an in-focus clear nucleolus and Nissl substance could be identified.

### Statistical Methods

For the serial *in vivo* MR spectroscopy data and viral loads, repeated measures analysis of variance (RM-ANOVA) in combination with Holm's t-tests was employed to isolate differences between time-points within the cohorts using JMP 7.0 (SAS, Cary, NC). For the *post-mortem* measures including IBA-1, SYN, MAP2, GFAP, neuronal counts and last MRS scans before sacrifice, ANOVA was performed among the cohorts and if found to be significant, Least Square Means Student's t-tests were used to isolate which cohort was significantly different. A p-value of ≤ 0.05 was considered to be significant.
